# Related differences in fecal bacteria of Chinese northern pregnant women of different ages: associations with maternal clinical indicators and neonatal outcomes

**DOI:** 10.3389/fmicb.2025.1642516

**Published:** 2025-07-18

**Authors:** Feifei Hu, Gaona Liu, Xin Sun, Yao Su, Mingli Huang

**Affiliations:** Department of Obstetrics, The First Affiliated Hospital of Harbin Medical University, Harbin, China

**Keywords:** pregnant women, age, clinical indicators, fecal bacteria, microbiota dysbiosis

## Abstract

The gut microbiota, a vital “microbial organ,” influences digestion, immunity, and metabolism. Aging alters gut microbiota of pregnant women through metabolic and hormonal pathways, thereby impacting neonatal health. In this study conducted in northern China, we compared two groups: advanced maternal age (AMA, ≥35 years) and younger maternal age (YMA, 20–34 years), analyzing fecal bacteria and maternal metabolism via biomarker measurements and microbial sequencing. Results showed AMA had significantly higher serum levels of alkaline phosphatase (AKP), 25-hydroxyvitamin D [25(OH)D], and creatinine, while YMA exhibited higher Cu but lower Fe concentrations. Although the fecal bacteria of AMA participants showed greater diversity, the YMA group displayed a more stable bacterial composition, characterized by a higher abundance of beneficial bacteria (e.g., *Bifidobacterium*) and a lower prevalence of potential pathogens (e.g., Streptococcus). Metabolically, the fecal bacterial network in YMA participants was more integrated, whereas the AMA group showed a “high-complexity, low-efficiency” pattern with disrupted metabolic pathways, which may contribute to adverse pregnancy outcomes. This study highlights age-related dysbiosis of the fecal bacteria in pregnant women and its impact on maternal and neonatal health, advocating for personalized prenatal care strategies for women with AMA.

## 1 Introduction

The gut microbiota, commonly referred to as the “second genome” of the human body, constitutes a complex ecosystem comprising trillions of microorganisms. These microbes establish a mutually beneficial relationship with the human host through intricate metabolic interactions, playing a critical role in physiological processes such as nutrient metabolism, immune regulation, and neural signaling (Afzaal et al., 2022; Gao, 2024). As a unique and vulnerable population, pregnant women experience profound alterations in their gut microbiota, influenced by factors such as pregnancy-induced physiological transformations, delivery methods, and postpartum recovery. Owing to differences in physical fitness, hormonal profiles, and lifestyle patterns across different age groups, the composition of the gut microbiota varies significantly (Huang et al., 2022; Xu et al., 2025). Thus, a comprehensive investigation into age-related structural alterations of the gut microbiota during pregnancy is essential for understanding maternal and neonatal health issues, while also facilitating the development of personalized health management strategies for women across life stages.

These physiological changes are intricately linked to the gut microbiota. During pregnancy, women undergo marked hormonal fluctuations—particularly elevated estrogen and progesterone levels-which profoundly impact intestinal microecological balance (Ma et al., 2024). Studies consistently demonstrate that both the diversity and composition of the gut microbiota in pregnant women undergo notable shifts (Song et al., 2024). For instance, relative abundances of major bacterial phyla (e.g., *Firmicutes* and *Bacteroidetes*) shift, with *Firmicutes* often becoming more predominant-a change likely associated with increased energy demands and adjustments in nutrient uptake during pregnancy. Concurrently, populations of beneficial bacteria (e.g., *Bifidobacterium* and *Lactobacillus*) fluctuate throughout gestation: some studies report declines in their abundance, while potential pathogens (e.g., *Enterobacteriaceae*) may increase. Such changes may elevate the risk of conditions like constipation and gestational diabetes in pregnant women (Acharya and Shetty, 2024).

Age-related structural variations in the gut microbiota are closely tied to maternal health during pregnancy and postpartum. Younger pregnant women typically exhibit a more stable and diverse gut microbiota, which supports optimal nutrient metabolism, reduces the risk of pregnancy complications (e.g., constipation, gestational diabetes), and accelerates postpartum recovery (Liu et al., 2025). In contrast, older pregnant women face challenges such as reduced intestinal epithelial cell renewal and impaired mucosal immunity, stemming from age-related declines in stem cell activity (Perler et al., 2023). Additionally, older women often have pre-existing health conditions (e.g., hypertension, diabetes), which disrupt the intestinal environment via impaired blood circulation and metabolic dysfunction. Frequent medication use during pregnancy to manage these conditions further exacerbates gut microbiota dysbiosis through pharmacological side effects (Erlin et al., 2025).

The maternal gut microbiota is transmitted to newborns via pathways such as delivery mode and breastfeeding, playing a pivotal role in infant intestinal development, immune system maturation, and neural development. A healthy gut microbiota in younger women transmits a greater abundance of beneficial bacteria to newborns, facilitating the establishment of a favorable intestinal microecosystem and supporting digestive/immune system maturation (Wang et al., 2024). Conversely, dysbiosis in older women’s gut microbiota may reduce beneficial bacteria transmission and increase pathogen transfer to newborns, disrupting normal gut microbiota colonization and elevating risks of neonatal infections, allergies, and other health issues (Liu et al., 2024). Current research on age-related gut microbiota changes has limitations: existing studies primarily describe microbiota structure, with limited understanding of the molecular mechanisms by which age influences composition (Li G. et al., 2023; Li M. et al., 2023). Additionally, there is a scarcity of research on microbiota structural differences across ethnic/geographical groups, and personalized strategies to modulate the gut microbiota require further exploration.

This study aims to compare and analyze basic clinical parameters and fecal bacteria structures between advanced maternal age (AMA) and younger maternal age (YMA) groups in northern China. It also investigates associations among maternal fecal bacteria, biochemical indicators, and neonatal physiological parameters. This research not only provides a theoretical basis for understanding the gut microbiota structure of local pregnant women but also offers valuable insights for comprehensive health management of older pregnant women and their newborns.

## 2 Materials and methods

### 2.1 Study population and sampling

A total of 40 pregnant women who underwent prenatal examinations at the First Affiliated Hospital of Harbin Medical University from June 2022 to May 2024 and delivered after 37 weeks of gestation were recruited. The inclusion criteria were as follows: (1) aged 20–45 years; (2) women who received prenatal care at the study hospital and were scheduled to deliver there; (3) women who provided written informed consent for blood sample donation during routine check-ups. Further inclusion criteria specified: pregnant women in late pregnancy (37 ≤ gestational weeks ≤ 42) planned to deliver at our hospital; primiparas or multiparas aged 20–45 years (corrected from 18–45 to align with prior criteria); Singleton pregnancy; No fever, diarrhea, or antibiotic use within 1 month before sampling; written informed consent for study participation. Exclusion criteria included: pregnant women with severe organic diseases (heart, brain, kidney, etc.); pre-existing diabetes, hypertension, immune/hematological/cardiovascular disorders; twin or multiple pregnancies; history of hemorrhagic or thromboembolic diseases; harmful lifestyle habits (smoking, alcohol/drug abuse); mental illnesses. Participants were categorized into two groups based on maternal age: advanced maternal age (AMA, ≥35 years) and younger maternal age (YMA, 20–34 years). The mode of delivery (vaginal vs. cesarean), maternal complications, and neonatal outcomes (Apgar scores, birth length, and weight) were systematically recorded and analyzed. This study was approved by the Ethics Committee of the First Affiliated Hospital of Harbin Medical University (Approval No. 2024JS67).

The body mass index (BMI) values of the pregnant women were calculated within 1 week prior to delivery, following the “Recommended Standards for Weight Gain During Pregnancy” newly released by China in 2022. The current classification criteria for adult BMI are defined as follows: normal range (18.5 ≤ BMI ≤ 23.9 kg/m^2^), overweight (24.0 ≤ BMI ≤ 27.9 kg/m^2^), and obese (BMI ≥28 kg/m^2^) (Sun et al., 2021).

Oral glucose tolerance test (OGTT): pregnant women consumed a normal diet for 3 consecutive days before the test and fasted for ≥8 h prior to examination. During OGTT, participants ingested 300 mL of a solution containing 75 g glucose orally within 5 min. Venous blood samples were collected at baseline, 1 h, and 2 h post-glucose intake. The blood glucose level should be lower than 5.1 mmol/L, 10.0 mmol/L and 8.5 mmol/L, respectively, before and 1 and 2 h after taking glucose. Gestational diabetes mellitus (GDM) was diagnosed if any blood glucose value met or exceeded the above thresholds.

### 2.2 Collection of serum samples and determination of biochemical parameters

A 2-mL fasting venous blood sample was collected from each participant within 1 week prior to delivery. Samples were centrifuged at 3,500 rpm for 15 min to isolate serum for subsequent analyses.

Serum concentrations of zinc (Zn), iron (Fe), copper (Cu), and calcium (Ca) were determined using a Cobas 701 fully automated biochemical analyzer (Roche, Basel, Switzerland). Aspartate transaminase (AST), alanine transaminase (ALT), and alkaline phosphatase (AKP) activities were measured using commercial kits from Nanjing Jiancheng Bioengineering Institute (Nanjing, China). The ALT/AST ratio was calculated to assess liver health (Cao et al., 2024). The concentration of 25-hydroxyvitamin D [25(OH)D] in serum was measured via fluorescence immunochromatography using a Savant-60 analyzer (Cusabio, Wuhan, China). The criteria for interpreting serum 25(OH)D levels were derived from the Clinical Practice Guidelines of the Endocrine Society. Specifically, a 25(OH)D concentration below 20 ng/mL was classified as deficiency, a concentration ranging from 20 ng/mL (inclusive) to less than 30 ng/mL was considered insufficient, and a concentration of 30 ng/mL or higher was regarded as sufficient. Serum creatinine (Scr) and hemoglobin (Hb) were analyzed using enzyme-colorimetric automated methods on a Modular Analytics EVO system (Roche, Neuilly-sur-Seine, France).

### 2.3 Collection of fecal samples

Fecal and blood samples from pregnant women were collected on the same day (1 week prior to delivery). Fecal specimens were obtained using sterile tubes, immediately placed in ice boxes upon collection, and stored at −80°C within 24 h until DNA extraction.

### 2.4 Fecal DNA extraction and 16S rRNA sequencing

Genomic DNA was extracted from fecal samples using the Omega M5635-02 Kit (Omega Bio Tek, Norcross, GA, United States), strictly adhering to the manufacturer’s protocol. The concentration and quality of the extracted DNA were precisely evaluated using a NanoDrop 2000 spectrophotometer (Thermo Fisher Scientific, Wilmington, DE, United States), while its purity was corroborated by agarose gel electrophoresis.

The V3–V4 hypervariable regions of the 16S rRNA gene were amplified with the 338F/806R primer pair (forward primer: 5′-CCTACGGGNGGCWGCAG-3′; reverse primer: 5′-GACTACHVGGGTATCTAATCC-3′). Subsequently, the amplicons were accurately quantified, pooled in equal amounts, and subjected to paired-end sequencing on the Illumina PE250 platform (Illumina Inc., CA, United States) at Majorbio Bio-Pharm Technology Co., Ltd. (Shanghai, China). The raw sequencing data were then deposited into the China National GeneBank DataBase, with the project ID registered as CNP0005435.

### 2.5 Analysis of 16S rRNA sequencing data

The paired-end (PE) reads generated from Illumina PE250 sequencing were initially merged according to their overlapping regions. Concurrently, stringent quality control measures were implemented, including sequence filtering, to ensure data integrity. Following sample demultiplexing, the remaining high-quality clean reads were clustered into operational taxonomic units (OTUs) at a 97% sequence similarity threshold. Subsequently, all OTUs were annotated using the Ribosome Database Project (RDP), and the read counts for each OTU were meticulously determined. Leveraging the OTU clustering results, a comprehensive suite of diversity indices was computed, and the sequencing depth was evaluated, as previously described by Huang et al. (2021). The relative abundances of bacteria, spanning from the phylum to the genus level, were accurately quantified using the Quantitative Insights into Microbial Ecology (QIIME) pipeline (O’Connor et al., 2023). To assess the within-group α-diversity, the Chao1, Shannon, and Simpson indices were calculated. For β-diversity analysis, principal components analysis (PCA) and principal coordinate analysis (PCoA) were performed on the unweighted UniFrac distance matrix. The results were visualized using a non-metric multidimensional scaling (NMDS) plot, as outlined by Donugama et al. (2021). The relative abundances of taxonomic groups at the phylum and genus levels for each sample were compared and presented in histogram format. Employing the gplots R package, heatmaps were generated to visualize bacteria with the highest relative abundances at the amplification sequence variant (ASV) level. Additionally, the linear discriminant analysis effect size (LEfSe) tool was utilized to identify taxa that showed significant differences between groups, facilitating the exploration of potential biomarkers and group-specific microbial signatures.

### 2.6 Statistical analysis

Data were processed using SPSS22.0 statistical software. Count data were expressed as *n*/% and the *χ*^2^ test was performed. Continuous variables are reported as ±s. After confirming the normal distribution through the normality test, one-way analysis of variance using the Tukey HSD test was applied to evaluate the differences between groups. A *p*-value of <0.05 was considered statistically significant. Spearman rank correlation analysis was used to examine the association between physicochemical parameters and the composition of the bacteria community. Data visualization and graphical display were completed using OriginPro 9.0 software, supplemented by R software for advanced multivariate analysis.

## 3 Result

### 3.1 Clinical information of subjects

The basic characteristics of the study cohort are shown in Table 1. BMI, a simple clinical index widely used to evaluate nutritional status, adiposity, and physical development, has garnered significant attention in clinical research due to its higher potential for intervention compared with non-modifiable factors (e.g., genetic or immune factors) (Nguyen et al., 2019; Sun et al., 2022). The age of the AMA group ranged from 35 to 41 years old, with an average age of 36.9 ± 1.71 years, while the age of the YMA group ranged from 27 to 34 years old, with an average age of 30.80 ± 2.24 years. The BMI of the AMA group ranged from 22.0 to 36.73 kg/m^2^, with an average BMI of 29.68 ± 4.28 kg/m^2^. The BMI of the YMA group also ranged from 24.47 to 39.65 kg/m^2^, with an average BMI of 28.40 ± 3.56 kg/m^2^. Notably, the BMI distribution showed greater variability in the AMA group compared with the YMA group.

**Table 1:** Basic characteristics of AMA and YAM.

Characteristics	AMA (*n* = 20)	YMA (*n* = 20)	*t*/*χ*^2^	*p*
Maternal characteristics				
Maternal age ( $\bar{\chi }$ ± s, year)	36.9 ± 1.71	30.80 ± 2.24	9.70	<0.001
BMI ( $\bar{\chi }$ ± s, kg/m^2^)	29.68 ± 4.28	28.40 ± 3.56	1.03	>0.05
Gestational diabetes mellitus (*n*, %)	6 (30)	1 (5)	4.33	>0.05
Gestational hypertension (*n*, %)	3 (15)	0 (0)	3.00	>0.05
Delivery mode (*n*, %)				
Cesarean (*n*, %)	19 (95)	16 (80)	1.43	>0.05
Vaginal (*n*, %)	1 (5)	4 (20)	1.80	>0.05
Infant characteristics				
Weight at birth ( $\bar{\chi }$ ± s, g)	3280.26 ± 716.94	3235.26 ± 498.74	0.23	>0.05
Length at birth ( $\bar{\chi }$ ± s, cm)	49.05 ± 2.46	49.58 ± 1.85	−0.78	>0.05
Apgar score ( $\bar{\chi }$ ± s)	8.52 ± 1.15	8.84 ± 0.37	−1.19	>0.05
Gender				
Male (*n*, %)	8 (40)	6 (30)	0.44	>0.05
Female (*n*, %)	12 (60)	14 (70)	0.44	>0.05

According to the “Standards for Recommended Weight Gain during Pregnancy” released by the National Health Commission of China in 2022. In the AMA group, 5 people (accounting for 25%) had overweight BMI (25.0 kg/m^2^ ≤ BMI < 28.0 kg/m^2^), and 13 people (accounting for 65%) were obese (BMI ≥28.0 kg/m^2^). In the YMA group, 6 people (accounting for 30%) had overweight BMI, and 9 people (accounting for 45%) were obese. As a special group, the impact of BMI on pregnant women is higher than that on the general population. Moreover, the BMI of pregnant women is closely related to gestational age and fetal weight, and also has a significant impact on pregnancy outcomes. The cesarean section rate of AMA was 95% (19/20), which was significantly higher than that of the YMA group (75%), with a significant difference (*p* < 0.05). And there was a significant positive correlation between the BMI of pregnant women and cesarean section. In the AMA group, 6 people (accounting for 20%) had gestational diabetes mellitus and 3 people (accounting for 15%) had gestational hypertension. In the YMA group, 1 person (accounting for 5%) had gestational hypertension. The incidence of gestational hypertension and gestational hyperglycemia in the AMA group was significantly higher than that in the YMA group (*p* < 0.05).

**Figure 1:**
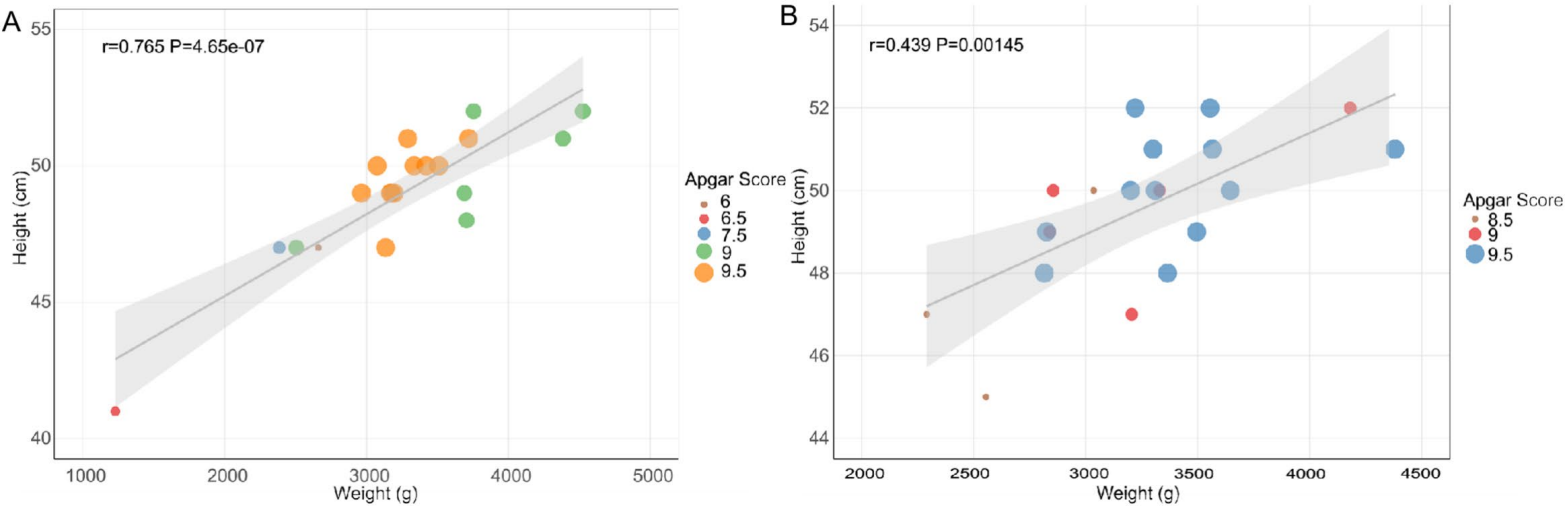
The linear regression scatter plots of height, weight and Apgar score of AMA **(A)** and YMA **(B)**.

This study investigates the correlation between maternal age and newborn outcomes by analyzing basic newborn indicators. The weight distribution of infants born to both YMA and AMA shows some overlap (Table 1). However, the weights of infants born to YMA predominantly cluster around 3,000 g, indicating a relatively concentrated distribution within the normal range with less variation. In contrast, infants born to AMA exhibit a broader weight range from 2,000 to 4,000 g, suggesting greater variability and an increased likelihood of extreme birth weights. The heights of infants born to women of YMA generally fall between 45 and 50 cm. While infants born to AMA also concentrate in this range, the lower limit is slightly lower, with some approaching 40 cm. This suggests a potential increase in growth-restricted infants with shorter stature in the AMA group. Newborns in the YMA group have Apgar scores between 8 and 10, indicating normal spontaneous breathing and circulation at birth. In contrast, some infants born to AMA have scores between 5 and 7.5, suggesting possible mild asphyxia requiring cardiopulmonary resuscitation measures. Data analysis reveals a significant impact of maternal age on the correlation between newborn height and weight (Figure 1). A strong linear correlation (*R* = 0.765, *p* = 4.65 × 10^−7^) is observed in the AMA group, with data points closely following the regression line. In contrast, the correlation is weaker (*R* = 0.439, *p* = 0.00145) in the YMA group, with increased dispersion and a flatter regression line. These differences may reflect more homogeneous pregnancy characteristics in AMA, such as standardized nutritional interventions or stricter prenatal care, which may strengthen the coordinated changes in growth indicators.

The serum characteristics of the study cohort are shown in Table 2. Vitamin D, an essential fat-soluble micronutrient in human physiology, exists primarily as 25(OH)D in circulation, which serves as the most reliable biomarker for assessing vitamin D status. The biologically active form of vitamin D plays a pivotal role in both fetal development and maternal calcium homeostasis (Karpova et al., 2022; Sadeghian et al., 2020; Varshney et al., 2023). Emerging evidence suggests that vitamin D insufficiency during gestation may contribute to numerous adverse outcomes across the perinatal continuum, including recurrent pregnancy loss, gestational diabetes mellitus, preeclampsia, intrauterine growth restriction, low birth weight, and various neonatal complications such as neurodevelopmental disorders, atopic conditions (including infantile eczema), and skeletal abnormalities with potential long-term consequences (Yu et al., 2023). The mean serum 25(OH)D level in the AMA group was 27.41 ± 2.80 ng/mL, with a sufficiency rate of 45% (9/20), an insufficiency rate of 30% (6/20), and a deficiency rate of 25% (5/20). Similarly, the YMA group exhibited a comparable mean 25(OH)D level (23.49 ± 1.12 ng/mL), yet with a lower sufficiency rate (25%, 5/20), a higher insufficiency rate (40%, 8/20), and a higher deficiency rate (35%, 7/20).

**Table 2:** Basic serum characteristics of AMA and YAM.

Maternal characteristics	AMA (*n* = 20)	YMA (*n* = 20)	*t*/*χ*^2^	*p*
25(OH)D ( $\bar{\chi }$ ± s, ng/mL)	27.41 ± 2.80	23.49 ± 1.12	5.81	<0.001
Scr ( $\bar{\chi }$ ± s, μmol/L)	42.76 ± 3.29	40.42 ± 1.32	2.95	0.005
Hb ( $\bar{\chi }$ ± s, g/L)	120.18 ± 8.41	118.35 ± 6.88	0.75	>0.05
ALT/AST ( $\bar{\chi }$ ± s, %)	1.48 ± 0.63	1.50 ± 0.36	−0.12	>0.05
AKP ( $\bar{\chi }$ ± s, U/L)	138.42 ± 8.73	166.80 ± 5.73	−12.15	<0.001
Zn ( $\bar{\chi }$ ± s, μg/dL)	8.44 ± 1.14	9.45 ± 1.36	−2.54	0.015
Fe ( $\bar{\chi }$ ± s, μg/dL)	14.50 ± 2.12	12.63 ± 2.37	2.63	0.012
Ca ( $\bar{\chi }$ ± s, μg/dL)	2.18 ± 0.14	2.21 ± 0.12	−0.73	>0.05
Cu ( $\bar{\chi }$ ± s, μg/dL)	22.58 ± 2.00	27.22 ± 3.83	−4.80	<0.001

Scr serves as a key biomarker for renal function evaluation. Elevated Scr levels or abnormal increments during pregnancy may indicate renal impairment, often associated with pregnancy-induced hypertensive disorders (e.g., preeclampsia) and acute exacerbation of chronic kidney disease. In this study, the AMA group exhibited a mean Scr level of 42.76 ± 3.29 μmol/L, ranging from 29.30 to 63.60 μmol/L, while the YMA group had a mean Scr level of 40.42 ± 1.32 μmol/L, ranging from 23.66 to 56.19 μmol/L (Table 2).

Hb measurement, a routine component of perinatal assessment, reflects both maternal physiological status and iron metabolism. Hb levels directly correlate with Fe stores, whereas excess Fe-acting as a potent pro-oxidant-may induce oxidative stress in pancreatic β-cells, potentially impairing insulin synthesis. In this study, the mean Hb concentration was 120.18 ± 8.41 and 118.35 ± 6.88 g/L in both AMA and YMA, with no statistically significant difference observed between the two groups, suggesting similar iron status despite age differences (Table 2). This similarity suggests that both groups maintained vigorous Hb function and reasonable nutritional iron intake, as no significant anemia risk was detected.

Pregnancy, a complex physiological process, involves dynamic fluctuations in estrogen and progesterone levels. These hormonal changes increase hepatic workload, thereby affecting tissue metabolism and inducing alterations in enzyme profiles, such as AST and ALT (Fan et al., 2024; Kushner et al., 2022). The average ALT/AST ratios for the AMA group and the YMA group are 1.48 ± 0.63 and 1.50 ± 0.36, showing no significant difference between the two groups (*p* > 0.05) (Table 2). However, the distribution of ALT/AST ratios is broader in the AMA group. Among pregnant women with gestational diabetes, the ALT/AST ratios reach 2.98 and 2.97, suggesting that elderly and gestational diabetes further influence metabolic processes in pregnant women.

AKP, a zinc-containing glycoprotein, hydrolyzes various natural and synthetic monophosphate esters in an alkaline environment (with an optimal pH of around 10) (Zaher et al., 2020). Its time-dependent increase during pregnancy indicates a critical role in placental function and fetal development (Arbib et al., 2021), influencing processes such as purinergic signaling, bone mineralization, and placental angiogenesis. The average AKP values for the AMA group and the YMA group are 138.42 ± 8.73 and 166.80 ± 5.73 U/L, with the YMA group showing higher values, potentially linked to fetal growth and development, maternal metabolism, liver function, and placental function (Table 2).

The concentrations of Zn, Fe, Cu, and Ca in the serum of AMA and YMA are presented in Table 2. The average concentrations of Ca in the AMA group were slightly lower than that in the YMA group. By contrast, the average concentrations of Zn and Cu were 8.44 ± 1.14 μg/dL and 27.22 ± 3.83 μg/dL, respectively, which were significantly lower than those in the YMA group (*p* < 0.05). In contrast, the average Fe concentration in the AMA group was 14.50 ± 2.12 μg/dL, significantly higher than in the YMA group (*p* = 0.012). Among the AMA group, individuals with gestational hypertension showed lower serum Ca levels. As multiple studies have indicated, imbalances in metal element levels during pregnancy may contribute to pregnancy complications.

### 3.2 Diversity of fecal bacteria

**Figure 2:**
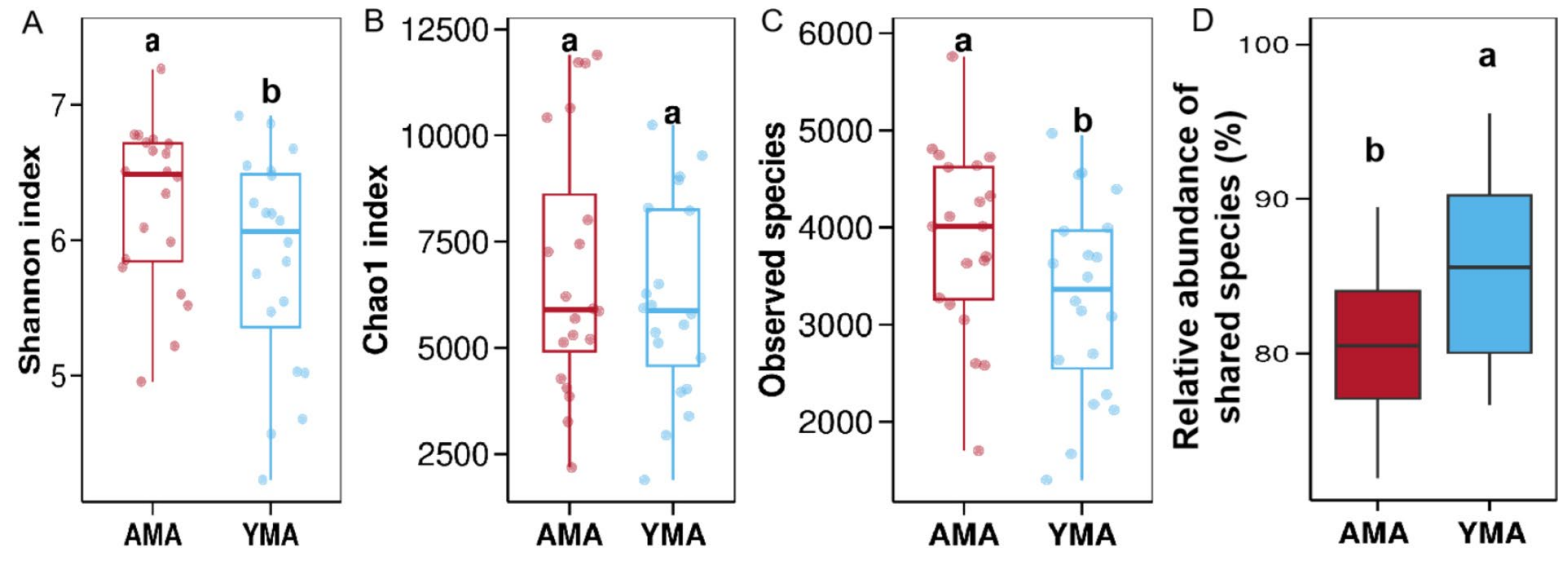
Analysis of diversity index differences in fecal bacterial communities between AMA and YMA groups. **(A)** Shannon’s diversity index. **(B)** Chao 1 index. **(C)** Observed species. **(D)** Inter-group differential OTUs.

All samples successfully underwent identification, DNA extraction, PCR amplification, and electrophoresis detection. The Rank-Abundance curves, Shannon-Wiener curves, and rarefaction curves of fecal bacteria in the AMA and YMA groups revealed distinct patterns. The AMA group’s Rank-Abundance curve declined more steeply and stabilized within a shorter horizontal range, indicating that a few bacterial species dominated the fecal flora-leading to uneven species distribution and a relatively small number of detectable OTUs. By contrast, the YMA group’s curve decreased gradually and extended further along the horizontal axis, suggesting a more even species abundance distribution without dominant bacterial species (Nothaaß and Huth, 2025). For the Shannon-Wiener curves, the YMA group exhibited consistently lower values across the entire sequencing range, reaching a plateau earlier with a lower Shannon index during the plateau phase. This suggests reduced species richness and evenness in YMA fecal bacteria, with limited addition of new species at deeper sequencing depths. In contrast, the AMA group maintained higher Shannon indices at equivalent depths, with significantly higher plateau-phase values, indicating greater species diversity. The rarefaction curves showed that the YMA group leveled off earlier after a certain number of samples, achieving a lower final cumulative OTU count-implying that few new species were discovered beyond a specific sample size and that YMA individuals had more similar bacterial compositions and a smaller overall species pool. Conversely, the AMA group’s curve rose more steeply, detecting more OTU types with the same number of samples; it continued to increase even after the YMA curve stabilized, requiring more samples to reach saturation and ultimately achieving a higher cumulative OTU count. This highlights greater inter-individual variation in AMA fecal bacteria, where each additional sample introduced numerous new species, leading to higher community richness. The α-diversity analysis results are presented in Figure 2. The Shannon index and Chao1 index of fecal bacteria in AMA were slightly higher than those in YMA. As key metrics for assessing bacterial diversity, a higher Shannon index reflects more even species distribution within the community, indicating greater bacterial diversity (Liu et al., 2025). The Chao1 index, which estimates species richness, suggests that larger values imply a higher actual number of species in the community (Shi et al., 2024). Collectively, these indices demonstrate that AMA fecal bacteria exhibit higher diversity and species richness than YMA. Additionally, the Observed species value was significantly higher in AMA (*p* < 0.05), indicating a greater variety of microorganisms under the same sampling conditions-highlighting aging as a significant factor influencing bacterial composition (Huang et al., 2022). The analysis of inter-group relative abundance differences, as shown in Figure 2D, reveals that the AMA group exhibited significantly lower relative abundance compared to the YMA group (*p* < 0.05). Specifically, the median relative abundance of core species in the AMA group was approximately 80%, while the YMA group exceeded 85%. Furthermore, the AMA group demonstrated greater inter-individual variability, as evidenced by a wider range and lower quartiles in the box plot, suggesting reduced bacteria sharing and increased community diversity. In contrast, the YMA group showed more consistent relative abundances and a more stable fecal bacterial structure. These findings indicate that maternal age influences the stability and uniformity of fecal bacterial communities, with AMA characterized by diminished bacterial sharing and enhanced diversity.

**Figure 3:**
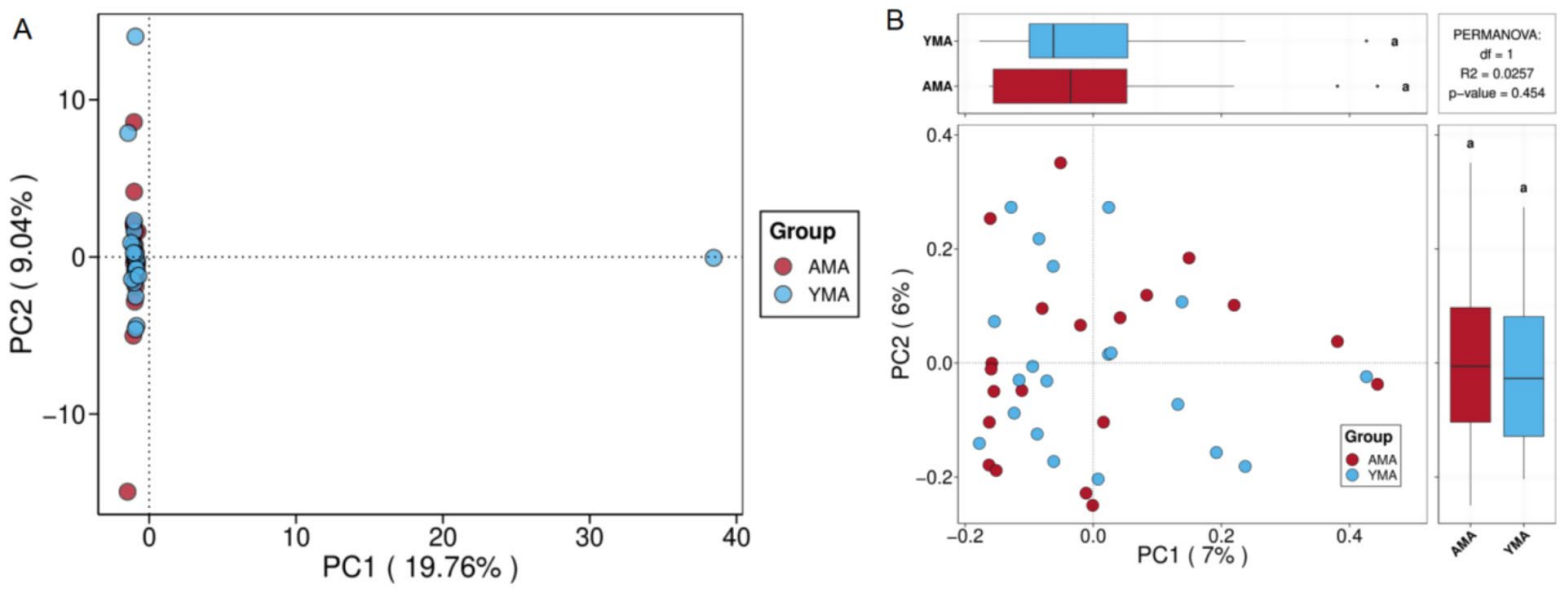
PCA score plot **(A)** and PCoA score plot **(B)** for fecal bacterial communities of AMA and YMA groups.

Figure 3A presented the PCA plot of fecal bacterial communities from AMA and YMA groups. PC1 and PC2 accounted for 19.76 and 9.04% of the total sample variation, respectively, with PC1 having exhibited higher explanatory power, which indicated good interpretability of the model. For inter-group differences dominated by PC1, samples from both groups were predominantly clustered in the negative axis region. In PC2-dominated differences, samples were distributed across all PC1 regions. The fecal bacterial community structures of the two groups showed no clear separation, as samples had significantly overlapped in both PC1 and PC2 dimensions, suggesting overall similar species compositions. Notably, an outlier sample in the YMA group was observed to display a distinct bacterial structure, potentially influenced by individual physiological or environmental factors. These findings suggested that maternal age had had a limited impact on fecal bacterial community structure. However, when combined with other analyses (e.g., α-diversity and core OTUs), it might have revealed differences in community diversity and stability.

PCoA is used to analyze similarities or differences in the composition of sample communities (Wang et al., 2025). As shown in Figure 3B, most samples from both groups clustered in the negative axis region of PC1. Within the main axis region of PC2, samples from both groups were evenly scattered, and substantial overlap was observed between the two groups in the principal coordinate axes (PC1 and PC2), indicating similar community compositions. The fecal bacterial community structures of the AMA and YMA groups showed no discernible separation trend. PERMANOVA analysis further corroborated this result, demonstrating no statistically significant difference between groups (*p* = 0.454, *R*^2^ = 0.0257), which suggested that maternal age had a limited impact on the overall structure of the fecal microecology in pregnant women. This finding indicated that although α-diversity and shared/unique species might differ between the two groups, β-diversity showed highly consistent overall community structures. Although the bacterial diversity and species richness in stool samples of the AMA group were numerically higher than those of the YMA group, the difference was not statistically significant, potentially attributed to the small sample size of this study.

### 3.3 Distribution of fecal bacterial communities

**Figure 4:**
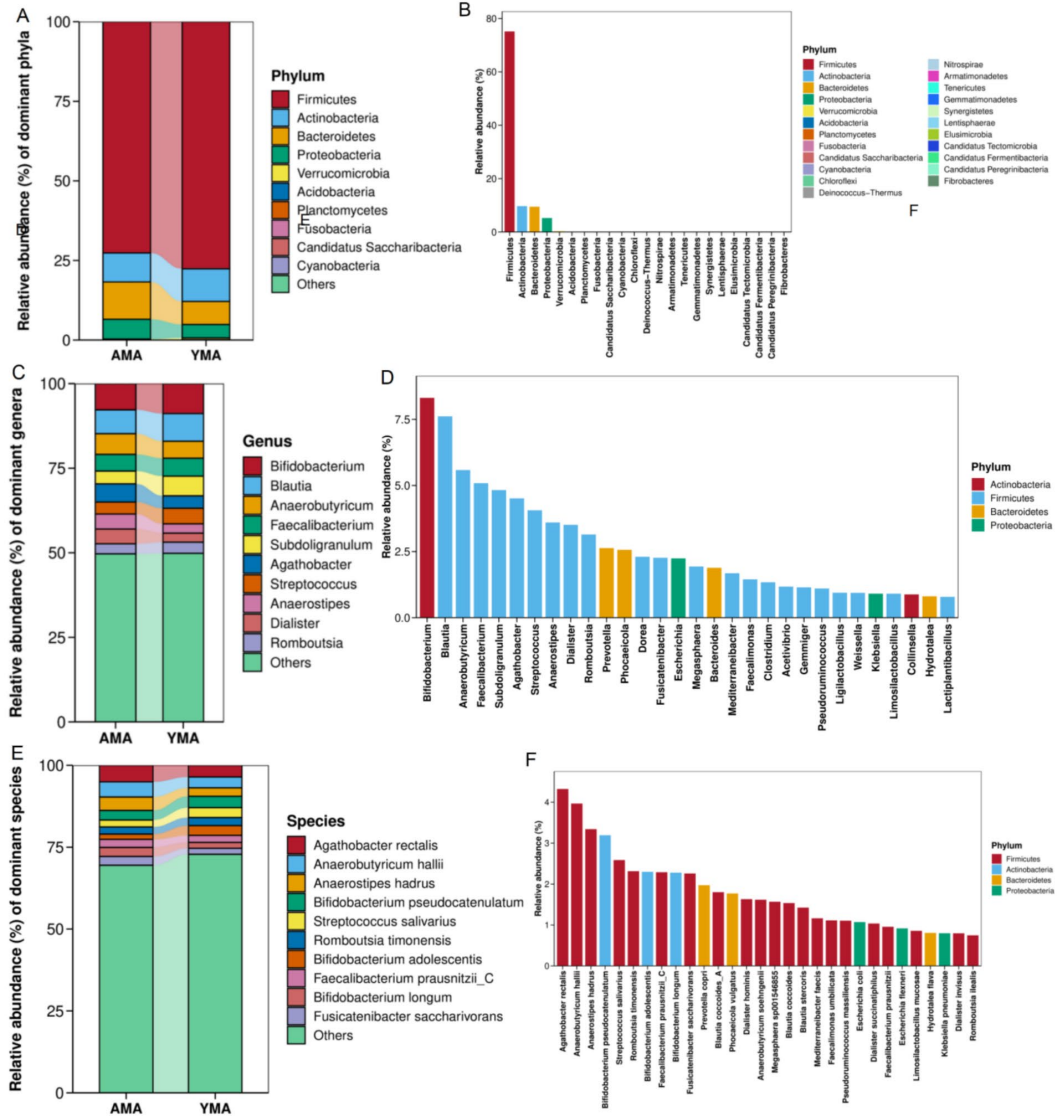
Accumulation maps of fecal bacterial community composition and dominant species maps for AMA and YMA groups at the phylum level **(A,B)**, genus level **(C,D)**, and species level **(E,F)**.

Figure 4 illustrates the differences in bacterial composition between the AMA and YMA groups at the phylum, genus, and species taxonomic levels. The fecal bacteria of pregnant women were predominantly composed of Firmicutes, with a relative abundance exceeding 80%. Firmicutes played a dominant role in maintaining the stability of the fecal bacterial structure and functional metabolism. The next most abundant phyla were Actinobacteria, Bacteroidetes, and Proteobacteria. Actinobacteria primarily included the genus *Bifidobacterium*, which has probiotic effects, while Bacteroidetes were closely associated with polysaccharide metabolism (Figures 4A,B). The AMA group exhibited higher relative abundances of Bacteroidetes and Proteobacteria compared to the YMA group. Conversely, the YMA group showed higher abundances of Firmicutes, Actinobacteria, Acidobacteria, and Cyanobacteria. Proteobacteria have known pathogenic roles in intestinal inflammation and colon cancer (Yang and Jobin, 2014). Additionally, low-abundance phyla such as Verrucomicrobia were exclusively detected in the AMA group, suggesting greater bacterial diversity in this subgroup.

At the genus level (Figures 4C,D), *Bifidobacterium* emerged as one of the predominant genera with the highest relative abundance across all samples, which showcased remarkable probiotic functions such as lactic acid production, immune regulation, and promotion of nutrient absorption. Notably, Freitas and Hill’s research has highlighted *Bifidobacterium*’s protective effects on the vaginal health of AMA, including reducing premature birth risks and enhancing the vaginal microbiome’s microecological balance (Freitas and Hill, 2017; Tabatabaei et al., 2019). *Bifidobacterium*, along with *Blautia*, *Agathobacter*, *Anaerobutyricum*, *Faecalibacterium*, and *Subdoligranulum*, constituted the most dominant genera in both groups. However, a notable finding was that the YMA group generally exhibited higher relative abundances of key probiotic genera, including *Bifidobacterium*, *Faecalibacterium*, and *Subdoligranulum*, compared to the AMA group. Conversely, the AMA group showed relatively higher abundances of potential opportunistic pathogenic genera, such as *Streptococcus*. The AMA group demonstrated significant enrichment in functional genera associated with short-chain fatty acid (SCFA) production, including *Agathobacter*, *Anaerobutyricum*, and *Anaerostipes* (Hijova and Chmelarova, 2007). In contrast, the YMA group exhibited generally lower abundances of these genera, suggesting a simpler and more stable bacterial structure. Notably, *Blautia*, *Faecalibacterium*, *Subdoligranulum*, *Anaerostipes*, and *Agathobacter* are prominent butyrate-producing genera within the *Firmicutes* phylum. These genera produce SCFA such as butyrate through fermentation, playing a crucial role in maintaining intestinal barrier function and anti-inflammatory environments. Additionally, *Prevotella* and *Bacteroides* within the *Bacteroidetes* phylum contributed significantly to bacterial diversity, participating in complex carbohydrate degradation and energy production (Accetto and Avguštin, 2021).

At the species level (Figures 4E,F), the dominant species in both samples primarily included typical butyrate-producing species such as *Agathobacter rectalis*, *Anaerobutyricum hallii*, *Anaerostipes hadrus*, *Faecalibacterium prausnitzii_C*, as well as probiotic *Bifidobacterium* species like *B. pseudocatenulatum*, *B. adolescentis*, and *B. longum*. These species played crucial roles in maintaining the stability of the host’s fecal microecology, promoting nutrient absorption, and regulating immunity. In the AMA group, various functional species such as *A. rectalis*, *F. prausnitzii_C*, *A. hadrus*, *A. hallii*, and *B. longum* were significantly enriched. Most of these species were butyrate producers or typical probiotics, contributing to intestinal anti-inflammation, metabolic regulation, and barrier protection. In contrast, the YMA group showed notably lower abundances of these key functional bacteria. *F. prausnitzii* and *A. rectalis*, as important anti-inflammatory probiotics, maintained abundances of great significance for health. Other species such as *Romboutsia timonensis* and *Fusicatenibacter saccharivorans* also demonstrated the diversity and complexity of the bacterial structure. Li G. et al. (2023) and Li M. et al. (2023) found that in patients with hyperlipidemic acute pancreatitis (HTGP), the abundances of *Bacteroides*, *F. prausnitzii*, and *B. uniformis* decreased, indicating severe complications and poor prognosis. This suggests that AMA might have enhanced their own immune capabilities by altering the composition of fecal bacteria. *A. rectalis* was a major dominant species shared by both groups, while the YMA group had a higher proportion of known beneficial species such as *F. prausnitzii_C* and *B. longum*, exhibiting good anti-inflammatory and immunomodulatory potential. In contrast, the YMA group had a higher relative abundance of non-core populations (others), possibly indicating a looser bacterial composition.

**Figure 5:**
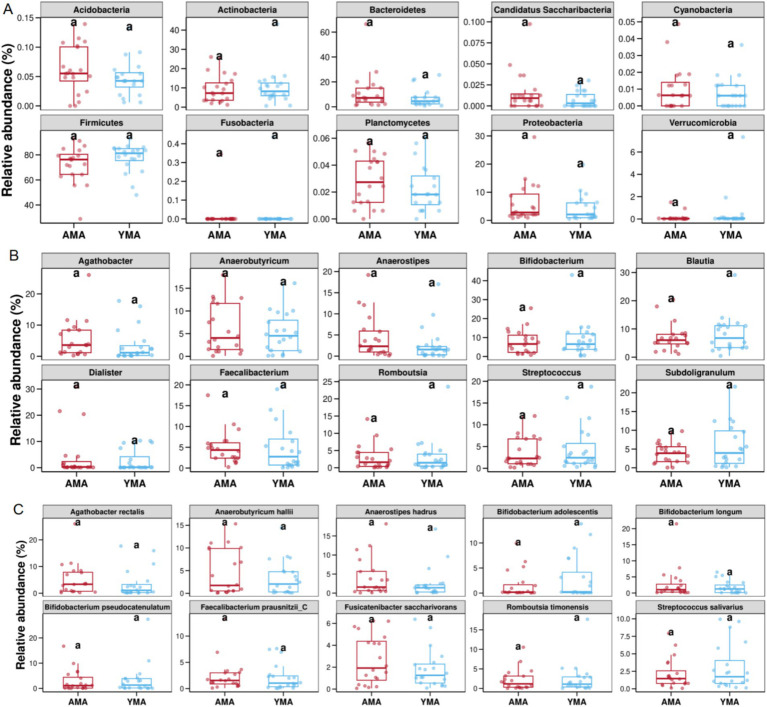
Top 10 differences in relative abundance of species among AMA and YAM (**A**, phylum level, **B**, genus level, **C**, species level).

At the phylum level, the top 10 differential phyla between the two groups were Acidobacteria, Actinobacteria, Bacteroidetes, Candidatus Saccharibacteria, Cyanobacteria, Firmicutes, Fusobacteria, Planctomycetes, Proteobacteria and Verrucomicrobia (Figure 5A). While relative abundance differences were not statistically significant, the AMA group had slightly higher average relative abundances of Acidobacteria, Bacteroidetes, Planctomycetes, and Proteobacteria, and slightly lower Firmicutes levels compared to the YMA group. The YMA group exhibited reduced bacterial diversity (α-diversity) and higher proportions of Proteobacteria and Actinobacteria, which might contribute to pregnancy-related inflammation. Additionally, a reduction was observed in butyrate-producing bacteria with anti-inflammatory properties (Koren et al., 2012; Mor et al., 2017).

At the genus level (Figure 5B), the top 10 differential genera were *Agathobacter*, *Anaerobutyricum*, *Anaerostipes*, *Bifidobacterium*, *Blautia*, *Dialister*, *Faecalibacterium*, *Romboutsia*, *Streptococcus*, and *Subdoligranulum*. Although relative abundance differences were not statistically significant, the AMA group had slightly higher average relative abundances of *Agathobacter*, *Anaerobutyricum*, *Dialister*, and *Anaerostipes*, and slightly lower levels of *Blautia* and *Subdoligranulum* compared to the YMA group. Notably, *Dialister* is enriched in the fecal bacterial of osteoporosis patients and may serve as a marker for spondyloarthritis.

At the species level (Figure 5C), the top 10 differential species were *Agathobacter rectalis*, *Anaerobutyricum hallii*, *Anaerostipes hadrus*, *Bifidobacterium adolescentis*, *Bifidobacterium longum*, *Bifidobacterium pseudocatenulatum*, *Faecalibacterium prausnitzii-C*, *Fusicatenibacter saccharivorans*, *Romboutsia timonensis*, and *Streptococcus salivarius* (Figure 5C). The AMA group had slightly higher average relative abundances of *A. rectalis*, *A. hallii*, *A. hadrus*, and *F. saccharivorans*, and slightly lower levels of *B. adolescentis* and *S. salivarius* compared to the YMA group. *B. adolescentis* and *S. salivarius* are well-known fecal probiotics that promote the growth of beneficial bacteria, inhibit harmful bacterial overgrowth, maintain fecal bacterial balance, and contribute to improved intestinal function and reduced inflammation.

### 3.4 Correlation analysis

**Figure 6:**
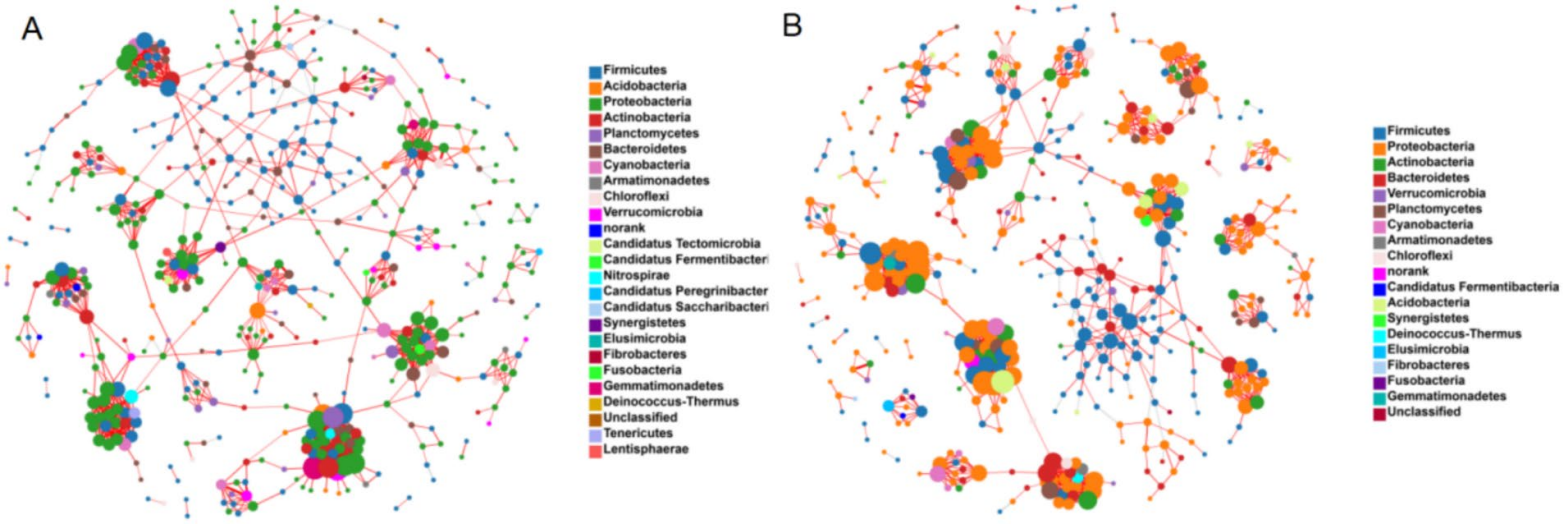
Co-occurrence of microorganisms in AMA **(A)** and YMA **(B)**.

The co-occurrence network analysis of bacterial communities revealed significant structural differences in the fecal bacteria between YMA and AMA groups (Figure 6). The network of YMA was composed of 465 nodes and 1,095 edges (network density: 0.01). Its short average path length (5.50) and high modularity (0.90) indicated that the community featured close connections and distinct functional partitions. In contrast, the network of AMA expanded to 489 nodes and 1,186 edges. Although the network density remained at 0.01, the average path length significantly increased to 7.17 (*p* < 0.05), and the network diameter decreased to 17.31, reflecting a reduction in the efficiency of information transfer between nodes and weakened network connectivity. Notably, the AMA group exhibited unique topological characteristics: the average clustering coefficient rose to 0.11 (compared with 0.09 in the YMA group), and the modularity slightly dropped to 0.88. Combined with its higher local clustered structure (clustering coefficient: 0.06 vs. 0.08), it was suggested that the fecal bacteria of AMA might adapt to physiological changes by forming more specific functional modules. However, this structural reorganization might undermine the overall network stability, potentially correlating with the observed increased risk of pregnancy complications.

**Figure 7:**
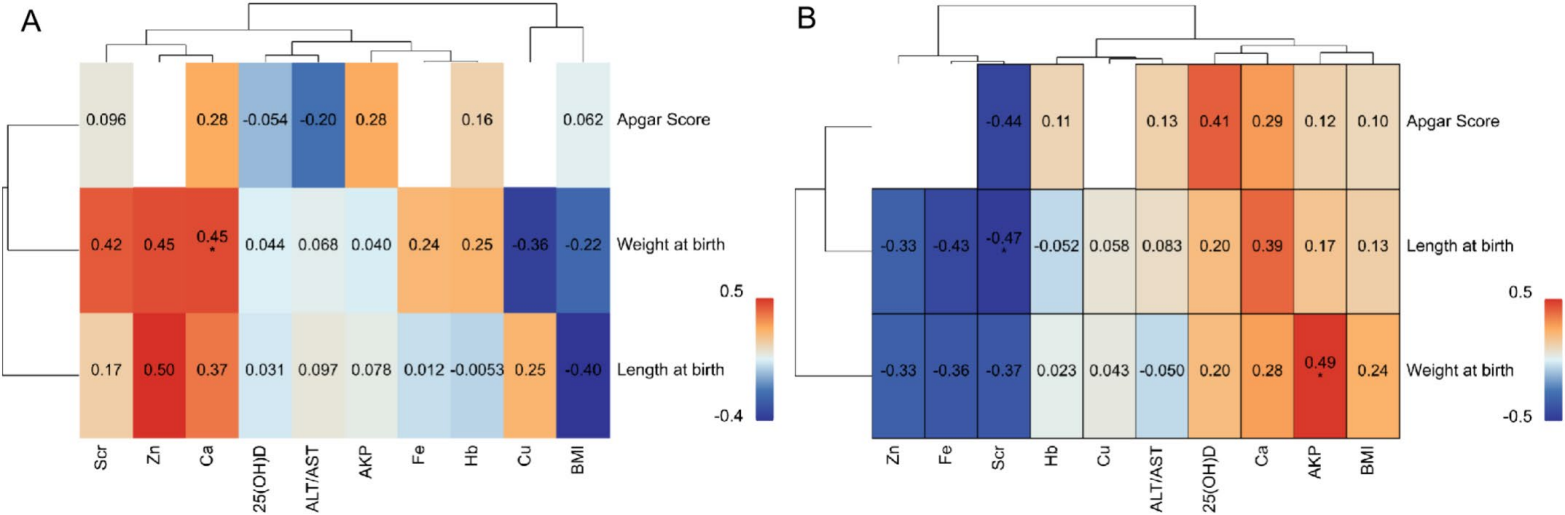
Correlation analysis of physiological indicators and neonatal indices for the AMA **(A)** and YMA **(B)** groups.

The correlation analysis between maternal blood biomarkers and neonatal health parameters revealed striking group-specific disparities (Figure 7). In the YMA group, Zn exhibited a positive trend with neonatal body weight (*p* = 0.50), while Ca showed a robust positive correlation with neonatal body length (*p* = 0.49). Notably, 25(OH)D not only demonstrated a significant positive association with body weight (*p* = 0.45) but also trended positively with body length (*p* = 0.25). By sharp contrast, in the AMA group, the positive correlation between 25(OH)D and neonatal weight completely dissipated (*p* = 0.13), while Zn showed a weakened positive tendency (*p* = 0.24) that failed to reach statistical significance. A particularly notable shift occurred for Fe: whereas the YMA group showed a positive trend with neonatal weight (*p* = 0.42), the AMA group exhibited a significant negative correlation (*p* = −0.44). Hb also displayed divergent patterns: a weak negative association with neonatal weight in the AMA group (*p* = −0.33) versus a positive trend in the YMA group (*p* = 0.25). For Cu, the YMA group showed a clear inverse relationship with neonatal length (*p* = −0.40), which markedly attenuated in the AMA group (*p* = −0.050). Scr demonstrated opposing associations: a positive trend with the Apgar score in the YMA group (*p* = 0.28) versus a significant negative correlation in the AMA group (*p* = −0.43). These systematic differences underscored the profound impact of reproductive age on maternal-fetal interconnections: key nutritional markers in the YMA group (notably Zn, 25(OH)D, Fe) exhibited synergistic effects on neonatal growth, whereas these beneficial associations were largely weakened or reversed in the AMA group-particularly the negative Fe metabolism trend and loss of 25(OH)D correlation. Concomitantly, metabolic waste markers like Scr showed enhanced negative effects in the AMA group. Collectively, these findings suggested that advanced maternal age pregnancies might involve compromised maternal-fetal nutrient transfer efficiency and heightened metabolic stress, potentially disrupting fetal developmental trajectories.

**Figure 8:**
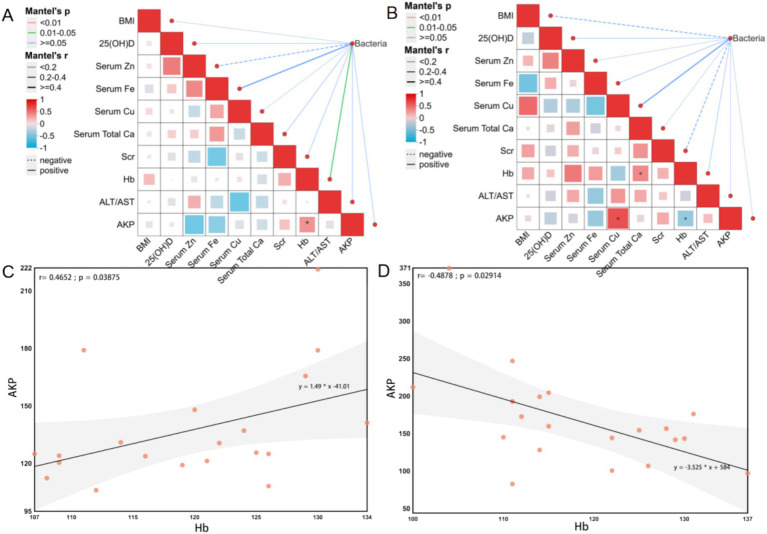
Mantel test of physicochemical indicators and bacterial communities for the AMA **(A)** and YMA **(B)** groups. Linear regression analysis of AKP and Hb in the blood of the AMA **(C)** and YMA **(D)** groups.

The Mantel test analysis in the YMA group revealed a metabolic interaction pattern significantly different from that in the AMA group (Figure 8). A strong positive correlation was observed between serum Cu and AKP (*R* = 0.8286, *p* = 0.0416) in YMA, suggesting that Cu metabolism may promote fetal bone mineralization by activating placental AKP activity, a mechanism that did not reach significance in the AMA group (*p* > 0.05). A positive association was observed between Hb and Ca (*R* = 0.4854, *p* = 0.0300) in YMA, indicating a synergistic effect between calcium homeostasis and erythropoiesis, while the strength of this association significantly decreased in AMA (*R* = −0.1811, *p* = 0.4448), suggesting that aging weakens the functional coupling of the calcium-hemoglobin metabolic axis. Comparisons between the two groups showed age-specific differences in the interaction between BMI and fecal bacteria: a weak negative correlation was observed between BMI and fecal bacteria in the YMA group (*R* = −0.2317, *p* = 0.96), and this trend was more significant in the AMA group (*R* = −0.3147, *p* = 0.997), but neither reached statistical significance, suggesting that body weight regulation may affect metabolic homeostasis through bacteria-independent mechanisms. The Fe metabolism network showed age-related reorganization: the negative correlation between serum Fe and Cu in the YMA group reflected the antagonism between Fe and Cu metabolism, while this association disappeared in the AMA group, possibly related to the imbalance of trace elements caused by increased oxidative stress in AMA. Significant differences were observed in liver and kidney function indicators: the critical positive correlation between Scr and Ca in the YMA group (*R* = 0.4204, *p* = 0.0649) suggested that renal function compensation may maintain homeostasis by regulating Ca and phosphorus metabolism, while the positive correlation between Scr and Hb in the AMA group (*R* = 0.3144, *p* = 0.1770) indicated that increased renal load may affect erythropoiesis. Regression model analysis (linear regression plot of AKP and Hb showed a significant negative correlation between serum AKP and Hb levels in the YMA group) (*R* = −0.4878, *p* = 0.0291), suggesting that enhanced bone marrow hematopoietic activity may inhibit erythroid cell production through a negative feedback mechanism. Notably, the AMA group showed an opposite correlation trend, which may be related to the decreased compensatory erythropoiesis ability caused by changes in the bone marrow microenvironment of AMA. Overall, the metabolic network of AMA exhibits the characteristics of “weak association-strong interaction,” and the dynamic balance of trace elements and organ function compensation are key nodes in maintaining pregnancy homeostasis.

**Figure 9:**
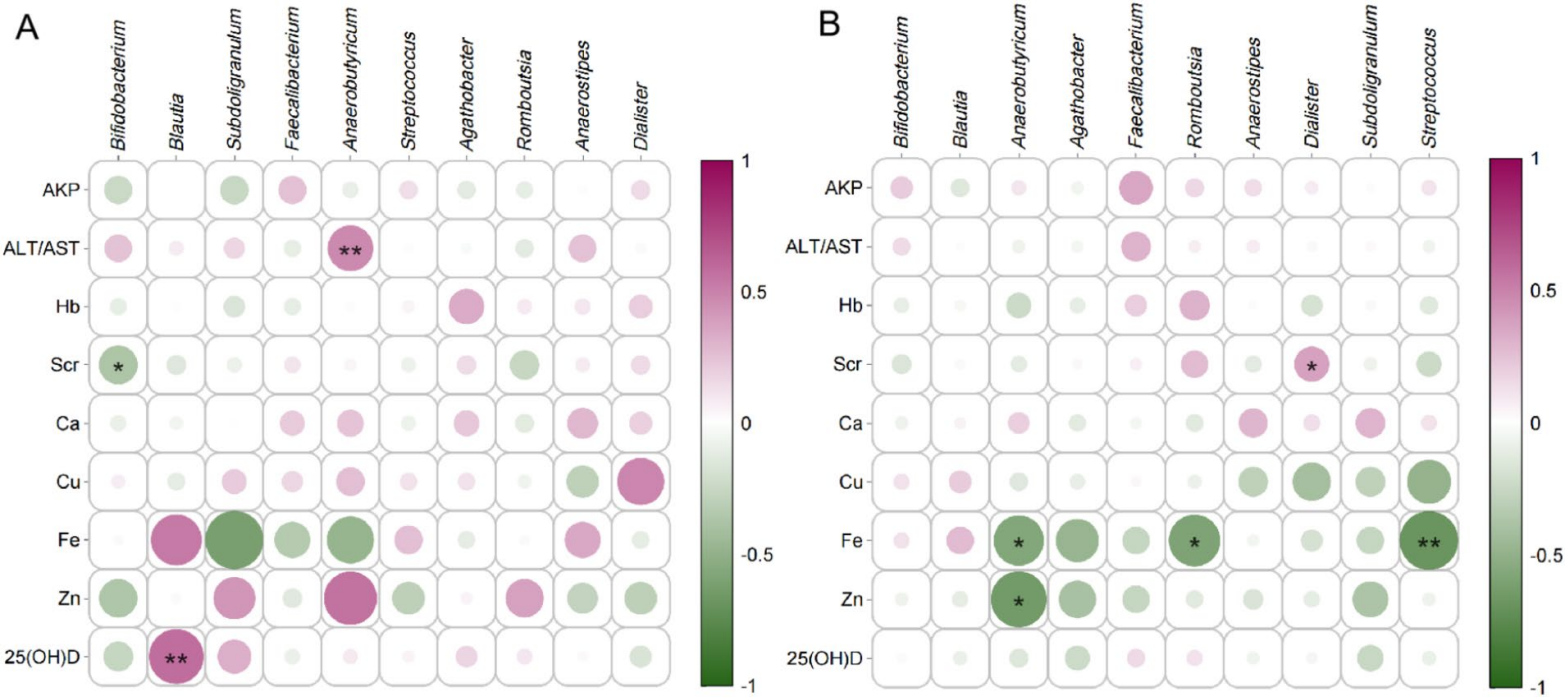
Correlation analysis between serum indices and major bacterial genera for the AMA **(A)** and YMA **(B)** groups.

The correlation analysis between physiological-biochemical blood indicators and key fecal bacteria in pregnant women is illustrated in Figure 9. *Bifidobacterium* exhibited a broader spectrum of significant positive associations in YMA, particularly with 25(OH)D levels. In AMA, the intensity of these positive correlations weakened notably, implying that the supportive role of this beneficial bacteria in regulating key metabolic markers may diminish with age. Another pivotal beneficial genus, *Faecalibacterium*, also demonstrated divergent trends across groups. In YMA, it showed moderate positive correlation tendencies with markers like AKP and certain mineral elements, whereas in AMA, it more frequently exhibited non-significant or slightly negative correlations-suggesting age-related shifts in its metabolic functional associations. Notably, mineral metal ions profiles revealed striking disparities: In YMA, Fe generally showed weak positive correlations with other bacterial genera, while in AMA, most genera (including *Blautia* and *Anaerobutyricum*) exhibited significant negative correlations with Fe. This strongly indicates a potential link between fecal bacteria composition in advanced age and systemic Fe metabolism/utilization disorders, warranting in-depth mechanistic investigation. *Blautia* displayed complex association patterns in both groups, but in AMA, its negative correlations with blood markers (e.g., Hb, Ca) were particularly pronounced. 25(OH)D-a critical biomarker-showed significant positive correlations with multiple genera (e.g., *Bifidobacterium*, *Romboutsia*) in YMA, but these robust associations nearly vanished or markedly attenuated in AMA, underscoring aging’s profound impact on the interaction between this key nutrient and intestinal microecology. Collectively, compared to YMA, AMA exhibited generally weakened or lost beneficial positive correlations between core fecal bacteria (notably *Bifidobacterium*, *Faecalibacterium*) and key blood parameters (especially 25(OH)D, Fe), accompanied by heightened negative associations-particularly with Fe. These systematic associative differences profoundly reflect that advancing reproductive age drives significant reconstruction of the dynamic network linking fecal bacterial and host physiological-biochemical status, suggesting a pivotal role for fecal bacteria in mediating the specific physiological traits and health risks of AMA.

## 4 Discussion

This initial microbial seeding significantly influences fetal development. Our study systematically measured clinical parameters of AMA and YAM, including serum creatinine levels, hemoglobin concentration, and serum metal ion content. These biomarkers provided direct insights into the metabolic profiles of pregnant women across different age groups. Concurrently, assessing neonatal physiological indicators such as body weight, length, and Apgar scores revealed that maternal age exerts a substantial impact on both maternal metabolism and neonatal health. We conducted a detailed comparative analysis of fecal microbiota between the two groups, aiming to elucidate the similarities and differences in species abundance and diversity. This aligns with prior research showing that pre-pregnancy BMI elevation increases risks of labor arrest, cesarean section, and macrosomia. AMA with higher BMI exhibit a heightened probability of gestational complications-including hypertension, hyperglycemia, instrumental delivery needs, postpartum hemorrhage, and abnormal neonatal birth weights (Sun et al., 2022). Metabolic dysregulation during pregnancy also contributes to preeclampsia (Davies et al., 2010; Rosinha et al., 2022), with gestational diabetes mellitus (GDM) incidence rising from 10.7% in women with BMI <23.9 kg/m^2^ to 23% in those with BMI ≥24 kg/m^2^. Pre-pregnancy obesity (BMI ≥30 kg/m^2^) significantly affects maternal gut microbiome. The proportion of Firmicutes (such as *Faecalibacterium*) increased, Bacteroidetes decreased, the diversity of microbiota decreased, and the abundance of SCFAs producing bacteria decreased. The deficiency of SCFAs weakened the intestinal barrier function, leading to lipopolysaccharide entering the blood and causing chronic inflammation. At the same time, the metabolic disorder of bacterial flora aggravates maternal insulin resistance and lipid abnormalities, promotes fetal overgrowth through the placenta, and is positively correlated with fetal abdominal circumference and fat accumulation.

YMA showed lower 25(OH)D levels, though AMA exhibited a wide distribution range; neither group achieved optimal sufficiency. Reduced skin synthesis and intestinal absorption with age, coupled with variable supplementation, may explain these trends. AMA displayed higher Scr levels due to age-related declines in glomerular filtration rate, impairing excretion. Elevated Scr correlated with increased proportions of newborns with birth weights <2,500 g and <1,500 g, consistent with reports linking maternal Scr elevation to preterm birth, low birth weight (LBW), and higher NICU admissions (Dvořák et al., 2021; Kendrick et al., 2015). Scr >60 μmol/L emerged as a critical threshold for adverse pregnancy outcomes (Harel et al., 2019). AMA with GDM showed Hb levels >130 g/L, aligning with first-trimester Hb’s positive correlation with subsequent GDM (Lee et al., 2023). YMA AKP activity, likely due to stronger placental function supporting fetal skeletal development (Manjunathappa et al., 2023). AMA displayed elevated serum Zn, with a positive correlation between Zn concentration and gestational hypertension risk. Zn’s role in over 200 enzymes and antioxidant defense contrasts with its paradoxical association with hypertension, while zinc deficiency is linked to immunity impairment and preterm birth (Ota et al., 2015). Maternal Ca absorption peaks in the second/third trimesters; Ca deficiency may drive vasoconstriction via parathyroid hormone release (Hillier et al., 2003). Cu, a superoxide dismutase component, showed lower levels in YMA, with early-pregnancy Cu deficiency associated with gestational hypertension (Lewandowska et al., 2019; Basu et al., 2015). Fe concentrations did not differ significantly between groups, though AMA with gestational hypertension showed elevated Fe (32.5 μg/dL vs. 28.51 μg/dL). This study underscores the interconnections between maternal age, metabolic biomarkers (e.g., Scr, Hb, 25(OH)D), and metal ion profiles (Zn, Cu, Ca, Fe) in modulating pregnancy outcomes. Elevated Zn and disrupted Cu/Fe homeostasis in AMA may contribute to gestational hypertension pathogenesis, warranting further mechanistic investigation.

The human gut microbiota represents a complex and dynamic community of microorganisms, including bacteria, viruses, and fungi, that coexist within the gastrointestinal tract. This microbiota begins to develop in early life and becomes more established in adulthood, influenced by factors such as age, ethnicity, antibiotic use, medications, and diet (Rubini et al., 2022). Bacteria within the gut microbiota perform essential functions, including food digestion, short-chain fatty acid production for energy and metabolic processes, immunomodulation, antibacterial protection, and the synthesis of vital vitamins, including intermediates of one-carbon metabolism. The Venn diagram data revealed that a total of 22,325 shared OTUs were identified between the AMA and YMA groups, demonstrating a high degree of consistency in the composition of the core fecal bacterial between these two groups. However, the AMA group exhibited a significantly higher number of unique OTUs (20,855) compared to the YMA group (14,653), suggesting greater inter-individual variability and increased microbial diversity in the fecal bacteria of AMA. This individualized microbiota profile may be closely linked to physiological or immunological changes associated with AMA, potentially making their fecal microecosystem more susceptible to external or host-related influences. In contrast, the bacterial communities in YMA group individuals showed greater stability and consistency, indicative of stronger bacterial balance and regulatory capacity. Although the α-diversity of the AMA group was numerically higher than that of the YMA group, β-diversity analysis showed that the fecal bacterial community structures of the AMA and YMA groups exhibited high overlap in PCoA, indicating consistency between the two groups at the community structure level. Overall, samples from the two groups formed independent clusters in the principal coordinate space, and their distribution areas did not separate significantly, underscoring minimal differences in bacterial structure and richness.

The AMA group exhibited more significantly enriched taxonomic units, reflecting that their fecal bacteria might tend to be diversified and heterogeneous due to age-related factors. This finding provided a basis for further exploration of the potential relationship between maternal age and fecal bacterial functions. The LDA discriminant analysis results revealed significant differences in multiple microbial taxonomic units between the AMA and YMA groups (Figure 9B). The YMA group exhibited enrichment of probiotic genera such as *Megasphaera*, *Lactiplantibacillus*, *Ligilactobacillus*, *Leuconostoc*, *Sellimonas*, and *Akkermansia*, which are involved in lactic acid and short-chain fatty acid synthesis. These genera play crucial roles in maintaining the intestinal mucosal barrier, regulating immune function, and promoting host metabolic health. Additionally, the YMA group showed significant enrichment of bacteria like *Capsulimonas*, *Desulfosporosinus*, *Erwiniaceae*, and *Acidaminococcus*, which are involved in nutrient metabolism, reflecting a more stable and functionally complete fecal microbiota structure. In contrast, the AMA group was enriched with multiple environmental-derived bacteria or potential opportunistic pathogenic genera, including Pseudomonas, Acinetobacter, Sphingobacterium, Flavobacterium, Moraxellaceae, Trichococcus and Sutterella. Furthermore, the AMA group exhibited enrichment of microbial groups associated with microbiota imbalance or immune response, such as *Verrucomicrobia_subdivision_3*, Weeksellaceae, Pedosphaera, and Holdemania. These findings suggested that the AMA group exhibited greater bacterial diversity but with a tendency toward functional dysregulation, potentially indicating an increased risk of ecological imbalance in their fecal bacterial ecosystem. Thus, maternal age may influence fecal bacterial structure and potentially affect microecological homeostasis and health status during pregnancy.

A healthy gut microbiota is characterized by a stable and balanced composition of major phyla, including Firmicutes and Bacteroidetes, with smaller contributions from Actinobacteria, Proteobacteria, and Verrucomicrobia (Ottman et al., 2012). Firmicutes are primarily involved in carbohydrate metabolism and energy extraction, while Bacteroidetes exhibit diverse functions, including polysaccharide degradation, immune system modulation, pathogen regulation, and detoxification. Actinobacteria, Proteobacteria, and Verrucomicrobia contribute to organic compound decomposition, immune support, and mucus degradation (Rinninella et al., 2019). Maintaining a healthy maternal fecal microbiota is crucial during pregnancy, as it impacts obstetric outcomes for both the mother and baby, as well as long-term health. Liu et al. (2017) reported that the gut microbiota of Chinese pregnant women was primarily composed of Firmicutes, Bacteroidetes, Fusobacteria, and Proteobacteria. They also observed that Bacteroidetes abundance in the gut microbiota of third-trimester pregnant women was higher than that of Firmicutes. However, our findings were the opposite: compared to YMA, the AMA group exhibited a lower proportion of Firmicutes. These results indicated that the fecal bacteria of pregnant women were highly concentrated at the phylum level, with Firmicutes as the predominant phylum, although its relative abundance was higher in the YMA group. The AMA group demonstrated a more diverse and balanced distribution of phyla, while the YMA group retained a certain degree of diversity and supplementary functional phyla. These findings suggested that aging might increase pathogenic factors in the gut, potentially leading to fecal bacterial imbalance. The YMA group exhibited higher diversity of functional genera at the genus level, which might contribute to maintaining intestinal homeostasis. This finding aligned with the research by Lee et al. (2022) and Şanlibaba et al. (2022), which demonstrated that advancing age typically resulted in decreased gut microbiota biodiversity and increased enrichment of opportunistic pathogens. Specifically, *Prevotella*, a Gram-negative anaerobic bacillus, was enriched in the AMA group and served as a dominant member of the human oral microbiome.

A human study showed that the core microbiota of elderly subjects differed from that of young adults, with a higher proportion of *Bacteroides* species. This result was consistent with our finding that the proportion of *Bacteroides* was higher in the AMA group (Claesson et al., 2011). The YMA group exhibited a more diverse microbiota composition and a higher proportion of probiotics at the phylum, genus, and species levels, reflecting a healthier and more stable fecal bacterial ecosystem. In contrast, the AMA group showed bacterial centralization, a lack of functional bacteria, and potential risks of ecological disturbance, indicating that maternal age may have a significant impact on the structure of fecal bacteria during pregnancy. However, the enrichment of functional bacteria might also be a compensatory manifestation of decreased microecological stability, suggesting that the age of pregnant women could affect their metabolic and immune homeostasis by regulating the composition of fecal bacteria. The AMA group exhibited a more intricate microecological composition. Aging was associated with reduced fecal bacterial diversity, a shift in dominant bacterial genera, and an increased relative abundance of potential pathogens, such as *Enterobacteriaceae* and *Staphylococcus*. In contrast, beneficial bacteria like *Bifidobacterium* and *Lactobacillus* were more abundant in the fecal samples of the YMA group.

The fecal bacteria of the AMA group might adapt to physiological changes by developing specialized functional modules. However, this structural reconfiguration potentially compromised the overall stability of the microbial network. Such instability may be linked to the elevated risk of pregnancy-related complications observed in this demographic.

The fecal bacterial communities of pregnant women at the phylum, genus, and species levels were characterized by the dominance of Firmicutes, richness of butyrate-producing bacteria and probiotics, and strong metabolic and immune functions, which provided a solid foundation for microecological homeostasis. This finding provided data support for further exploration of microecological differences between AMA and YMA and their potential health impacts. Fecal bacterial analysis revealed a strong positive correlation between bacterial abundances and serum Cu in the YMA group (*R* = 0.6428, *p* = 0.1264), approaching the significance threshold, suggesting that bacteria might affect metabolism by regulating copper absorption. However, this association was not observed in the AMA group (*R* = −0.1887, *p* = 0.698), indicating that aging led to the degradation of the interaction network between bacteria and trace elements. These findings collectively indicated that the metabolic network of YMA had stronger functional integration, while the AMA group exhibited characteristics of “a disorganized metabolic network with reduced functional efficiency.” The decoupling of key metabolic pathways might be an important mechanism leading to adverse pregnancy outcomes in AMA. Although this study initially explored the characteristics of the fecal bacteria of pregnant women of different ages, the single-center design had a limited sample size, which might have affected the universality of the research results. For future work, based on the initial insights from the current data, recruitment will be expanded across multiple centers to validate and expand these findings in more diverse populations. Potential biases (e.g., regional, demographic, or clinical practice differences) will be eliminated through large multicenter studies, enhancing the accuracy and external validity of the results.

## 5 Conclusion

This study systematically measured the clinical parameters and fecal bacteria dysbiosis between AMA and YMA groups, and explored the characteristic differences and correlations of fecal bacteria in pregnant women of different ages by evaluating physiological indicators of newborns such as body weight, body length, and Apgar score. In the AMA group, fecal bacteria diversity was reduced, the ratio of Firmicutes to Bacteroidetes was imbalanced, and the abundance of potential pathogens such as *Enterobacteriaceae* was increased, while probiotics such as *Bifidobacterium* were more enriched in the YMA group. In terms of metabolism-microbiota interaction, the correlation between serum metal ions (such as Cu and Fe) and fecal bacteria was weakened in the AMA group, suggesting that aging may interfere with the coupling effect of the microbe-metabolism network. Regarding neonatal health correlation, maternal fecal bacteria diversity was positively correlated with neonatal body weight and Apgar score, while fecal bacteria imbalance in the AMA group may be associated with an increased risk of adverse neonatal outcomes. Through multi-dimensional analysis, this study reveals the remodeling effect of maternal age on intestinal microecology and its potential association with pregnancy metabolism and neonatal health, providing a theoretical basis for microecological intervention in AMA pregnancy.

## Data Availability

The original contributions presented in the study are publicly available. This data can be found at: https://www.ncbi.nlm.nih.gov/sra, accession number SRP456518.
